# Amyloid burden identifies neuropsychological phenotypes at increased risk of progression to Alzheimer’s disease in mild cognitive impairment patients

**DOI:** 10.1007/s00259-018-4149-2

**Published:** 2018-09-22

**Authors:** Andrea Ciarmiello, Antonio Tartaglione, Elisabetta Giovannini, Mattia Riondato, Giampiero Giovacchini, Ornella Ferrando, Marina De Biasi, Chiara Passera, Elena Carabelli, Antonio Mannironi, Franca Foppiano, Bruno Alfano, Luigi Mansi

**Affiliations:** 1 Nuclear Medicine Department, S. Andrea Hospital, 19124 La Spezia, Italy; 2 Memory Laboratory CNS-ONLUS, 19124 La Spezia, Italy; 3 Medical Physics Department, S. Andrea Hospital, 19124 La Spezia, Italy; 4 Department of Neurology, S. Andrea Hospital, 19124 La Spezia, Italy; 5 Biostructure and Bioimaging Institute, National Research Council, 80145 Naples, Italy; 6 Interuniversitary Research Center for Sustainable Development (CIRPS), 00038 Rome, Italy

**Keywords:** Mild cognitive impairment, PET imaging, Beta-amyloid, Cognitive trajectory, Memory performance

## Abstract

**Purpose:**

The extent of amyloid burden associated with cognitive impairment in amnestic mild cognitive impairment is unknown. The primary aim of the study was to determine the extent to which amyloid burden is associated to the cognitive impairment. The secondary objective was to test the relationship between amyloid accumulation and memory or cognitive impairment.

**Materials and methods:**

In this prospective study 66 participants with amnestic mild cognitive impairment underwent clinical, neuropsychological and PET amyloid imaging tests. Composite scores assessing memory and non-memory domains were used to identify two clinical classes of neuropsychological phenotypes expressing different degree of cognitive impairment. Detection of amyloid status and definition of optimal amyloid ± cutoff for discrimination relied on unsupervised k-means clustering method.

**Results:**

Threshold for identifying low and high amyloid retention groups was of SUVr = 1.3. Aß + participants showed poorer global cognitive and episodic memory performance than subjects with low amyloid deposition. Aß positivity significantly identified individuals with episodic memory impairment with a sensitivity and specificity of 80 and 79%, (χ2 = 21.48; *P* < 0.00001). Positive and negative predictive values were 82 and 76%, respectively. Amyloid deposition increased linearly as function of memory impairment with a rate of 0.13/ point of composite memory score (*R* = −44, *P* = 0.0003).

**Conclusion:**

The amyloid burden of SUVr = 1.3 allows early identification of subjects with episodic memory impairment which might predict progression from MCI to Alzheimer’s disease.

**Trial registration:**

EudraCT 2015-001184-39.

## Introduction

Mild cognitive impairment (MCI) identifies the transitional stage between normal neurocognitive ageing and the progression towards several subtypes of dementia, including Alzheimer’s disease (AD) [1]. To date, there appears to be no single underlying neuropathological condition characterizing MCI, and indeed the clinical syndrome of MCI features a broad spectrum of subtypes, which differentiate from one another based on their underlying aetiology into AD, fronto-temporal dementia, vascular cognitive impairment, dementia with Lewy bodies, Parkinson’s disease, Huntington’s disease, HIV/AIDS, traumatic brain injury and substance abuse [2].

During recent decades, numerous efforts have been made to identify clinical markers to be used as reliable predictive markers of disease progression and thus collecting individuals at increased risk to develop AD. To date there are no means to accurately identify those likely to progress from MCI to advanced stages of dementia. Nor, in the latter case, is there a way to establish the aetiology and the pathophysiology of the process responsible for conversion, whether it be AD or other dementing conditions. Similarly, the well-established distinction among amnesic (aMCI), non amnesic (naMCI) or multiple domain MCI (mdMCI) although useful from a clinical point of view does not help to identify MCI converters to AD [3].

The recent availability of different biomarkers provide an additional tool in the definition of the pathological process. According to the evidence accumulated so far the progression from MCI to AD is characterized by a cascade of functional and structural brain and cerebral-spinal fluid (CSF) changes, starting many years before the AD onset, during which biomarkers become sequentially abnormal without clinical evidence of dementia. To date, the most convincing model of progression of cognitive impairment is that offered by Jack and colleagues in which CSF Aβ42 and brain amyloid biomarkers are the first to become abnormal, followed by biomarkers of neurodegeneration, before symptoms of AD become clinically detectable [4]. The dynamic model described by Jack and colleagues has been confirmed by a longitudinal study on dominantly inherited Alzheimer’s subjects that demonstrated the temporal progression of biomarkers changes which are characterized by an early tau and amyloid deposition increase followed, in time sequence, by neuronal dysfunction and neurodegeneration as measured by ^18^F- Fluorodeoxyglucose (FDG)-PET and magnetic resonance imaging (MRI), respectively [5].

High levels of amyloid deposition in the brain, also referred to as amyloid positivity, are associated with an increased risk of cognitive impairment and progression to advanced stages of dementia [6, 7]. Moreover, amyloid positive subjects are reported to progress at a faster rate towards neurologic degeneration and cognitive impairment [7–9]. Amyloid positivity is generally defined as a value of tracer uptake expressed as standardized uptake ratio (SUVr) measured in specific brain regions (such as dorsolateral prefrontal, ventrolateral prefrontal, orbitofrontal, superior parietal, lateral temporal, lateral occipital, and cingulate cortex) exceeding a predefined threshold value on amyloid PET imaging.

While threshold-based amyloid positivity detection may support the diagnostic work-up when the population is represented by two distinct classes of healthy and diseased subjects, it may also lead to erroneous classification when evaluating heterogeneous patient classes like MCI featuring intermediate characteristics between “normals” and demented.

Moreover, the assessment of a threshold based on AD population which is characterized by high levels of amyloid retention moves the value upwards, which can be useful when the primary aim is to reduce the number of false positive cases, but may be unable to identify MCI subjects with a slight amyloid deposition levels already associated with cognitive impairment or memory impairment [10].

In order to evaluate the level of amyloid deposition associated with early signs of cognitive impairment, the study investigated a sample of amnestic MCI spanning across different degree of cognitive impairment. Subjects underwent neuropsycological assessment and amyloid PET imaging with [^18^F]Florbetaben, a radiopharmaceutical already approved for detecting brain cortical amyloid deposition [11].

The primary aim of the study was to determine the extent at which amyloid burden is associated to the cognitive impairment as assessed by neuropsychological tests.

The secondary objective was to test the relationship between amyloid accumulation and memory or cognitive impairment.

## Methods

The present prospective cross-sectional study was conducted at two institutions, the S. Andrea Hospital and Mem Lab & Clinics in La Spezia (Italy) between December 2015 and June 2017. The Nuclear Medicine and Neurology units of S. Andrea Hospital were involved in patient recruitment, clinical evaluation and PET imaging, and Mem Lab & Clinics conducted neuropsychological assessment.

### Patients

The study enrolled a total of 66 participants, age ≥ 50 years, based on their medical history, clinical manifestations, and neuropsychological assessment.

Subjects inclusion criteria according to Petersen definition for amnestic MCI (aMCI) [12] consisted of Mini-Mental State Examination (MMSE) uncorrected score ≥ 24, Clinical Dementia Rating (CDR) of 0.5, absence of dementia and preserved basic activities of daily living (ADL) [13].

Presence of diseases potentially related to memory impairment, such as normal pressure hydrocephalus, Parkinson’s disease, or progressive supranuclear palsy, major structural abnormalities, signs of major vascular pathology such as intracerebral aneurysm or arteriovenous malformation, infarction, extensive leucoencephalopathy were among the exclusion criteria which also included relevant ischemic processes causing cognitive impairment, in accordance with the NINDS–AIREN criteria [14], clinical history of depression within the past year, ongoing treatment with psychotropic medication (e.g., antidepressants, neuroleptics), drug consumption and alcohol abuse.

### Standard patient consent, protocol approvals, and registrations

All participants gave written informed consent after a complete written and verbal description of the study. The study had been previously approved by the regional medical ethics committee, authorized by the Italian Competent Authority (AIFA) and registered in the EudraCT database as non-profit phase III clinical trial (EudraCT number 2015–001184-39).

### Clinical assessment

All participants underwent neurologic examinations, neuropsychological assessment and [^18^F]-Florbetaben PET/CT scan. The age of onset of the first signs of cognitive impairment was tracked back by means of a semi-structured interview to family members. MMSE scores, used for the statistical analysis as a measure of global cognitive status [15], were corrected for age and education levels according to the Italian norms (MMSEc) [16].

In this study MMSE was used as an index of global cognitive performance to identify the most impaired subjects. Among MCI subjects, those with lower cognitive performance were classified as aMCI+ (MMSEc<=24), whereas study participants with MMSEc>24 were defined as aMCI– [12–15]. Clinical severity was determined using the Clinical Dementia Rating (CDR) scale [17].

### Neuropsychological assessment

Neuropsychological evaluation included neuropsychiatric interview and a comprehensive battery of cognitive tests carried out within 2 weeks prior to PET scan by certified clinical psychologists, who were blinded to the subjects’ cognitive status.

Participants were administered the following tests: Prose Memory Test (PR) [18], Rey-Osterrieth Complex Figure Test – Copy (RFCTc) [19, 20] Rey-Osterrieth Complex Figure Test - Recall (RCFTd) [20], Category Verbal Fluency (CVF) [18], Digit Symbol Substitution Test (DS) [21, 22], Digit Span forwards (DSf) and backwards (DSb) [23].

Individual scores of each test were Z-transformed with reference to the mean and SD of the whole sample. Results were grouped into Episodic Memory Composite scores (EMCs) and Non-Memory Composite scores (NMCs). Individual EMCs was expressed by averaging Z-score of RCFTd and PR and individual NMCs was the average of Z-scores for RFCTc, CVF, DS, DSf and DSb [24].

### PET imaging and preprocessing procedures

PET/CT images were acquired in 3D mode 86 ± 8 min after intravenous injection of 306 ± 29 MBq of [^18^F]Florbetaben (FBB) (Neuraceq™) on a DISCOVERY TM 710 PET/CT scanner (GE Medical Systems. Milwaukee, WI, USA). PET projection data were iteratively reconstructed using 3-D OSEM algorithm of 8 iterations, 48 subsets, postsmoothed by a Gaussian filter of 3 mm FWHM, and with CT based attenuation correction. Image processing were performed using SPM12 (http://www.fil.ion.ucl.ac.uk/spm/software/SPM12) implemented under Matlab 8.6 (MATLAB R2015b, Mathworks Inc., Natick, MA, USA).

PET/CT images were spatially normalized to standard atlas coordinates in Talairach space using SPM T1 template [25].

PET data were converted to standardized uptake values (SUV) by scaling each image according to the body weight of each subject to the injected dose. Standardized Uptake Value Ratio (SUVr) was generated by dividing all regional SUV by the cerebellar gray matter SUV. For each subject, grey, white matter and cerebrospinal fluid (GM, WM, CSF) compartments were segmented from CT images using the segmentation routine implemented under SPM12 [26].

GM and WM voxels were then labeled according to their location, by use of Talairach Daemon database [27]. For the purpose of this study, we defined six volumes-of-interest (VOI): frontal (including inferior, medial, middle and superior gyrus), parietal (superior, inferior lobe, angular and supramarginal gyrus), temporal (inferior, middle, superior and parahippocampal gyrus), occipital (middle and inferior gyrus), posterior cingulate, and cerebellum. These VOIs were transferred onto the corresponding PET dataset to calculate the SUV mean of each brain region. Amyloid cortical burden (Aß burden) was calculated as the average SUVr of the area-weighted mean for frontal, parietal, temporal, occipital and cingulate VOIs.

### Statistics

Data were analyzed with the JMP statistical software package (SAS, Institute; Cary, NC, USA).

Individual with EMCs or NMCs lower than 10th percentile of positive values (i.e. EMCs and NMCs ≥0) were considered abnormal. Based on the value of Aß burden, subjects with high Aß tracer deposition (Aß+) were set apart from those with low Aß deposition (Aß-) by applying the k-means cluster analysis. This method is used for a priori classification of subjects in different groups by calculating the centroid for each group and assigning each subject to the group with the closest centroid [28, 29]. The optimal cut-off to separate Aß + from Aß- was SUVr = 1.30 and corresponded to 90th percentile of Aß- cluster [29].

The analysis was restricted to two clusters representing Aß + or Aß-patients whose cognitive performance were analyzed both in terms of single test results and Z-trasformed EMCs and NMCs.

A linear regression model was used to evaluate the relationship between cortical amyloid deposition and global cognitive, memory and non-memory performance. The slope of regression was used to estimate the rate of amyloid deposition associated to cognitive changes.

Chi-square analyses used to test the extent to which amyloid positivity increases the risk of episodic memory, non-memory cognition and global cognitive performance impairment. Odds ratios (ORs) and their 95% confidence intervals (CIs), positive predictive value (PPV), and negative predictive value (NPV) were calculated to assess the magnitude of associations.

The differences between the mean score of neuropsychological tests in amyloid positive and negative groups were determined with one-way ANOVA.

Continuous data were analyzed using independent t-tests, with degrees of freedom adjusted for inequality of variance where appropriate. For all tests, significance was assumed as *P* < 0.05.

## Results

Of the 66 participants initially recruited, three patients were excluded due to protocol deviations or to head movement that did not allow images analysis, thus leaving 63 subjects (who completed neuropsychological evaluation and PET imaging) for data analyses. As expected, the study sample included a slightly higher percentage of women (Table 1).

**Table 1:** Subject characteristics

Characteristic	All subjects	Aß+	Aß−	F-value	*P*
Demographic data					
N	63	34	29		
Male	27 (63)	13 (34)	14 (29)		
Female	36 (63)	21 (34)	15 (29)		NS*
Age, years	75.97 ± 6.59	76.38 ± 6.03	71.52 ± 7.98		0.01°
Education, years	9.97 ± 4.24	9.97 ± 4.01	10.69 ± 3.92		NS°
Neuropsychological battery					
CDR	0.5 ± 0	0.5 ± 0	0.5 ± 0		
Rey-Osterrieth Figure Copy	28.68 ± 7.86	22.63 ± 10.51	31.34 ± 5.58	16.05	0.0002^
Rey-Osterrieth Figure Recall	11.96 ± 5.04	10.55 ± 4.61	15.09 ± 5.73	12.12	0.0009^
Prose Memory	8.55 ± 3.26	6.71 ± 3.51	9.98 ± 2.84	16.50	0.0001^
Category Verbal Fluency	16.92 ± 4.78	15.04 ± 4.6	18.14 ± 5.95	5.44	0.02^
Digit Symbol	7.64 ± 2.1	6.29 ± 2.42	8.66 ± 2.58	14.02	0.0004^
Digit Span forwards	4.83 ± 1.07	5.04 ± 1.07	5 ± 0.92	0.02	NS^
Digit Span backwards	3.75 ± 0.86	3.75 ± 0.98	3.86 ± 0.86	0.22	NS^
MMSE	25.4 ± 3.07	24.74 ± 3.52	26.16 ± 2.27	3.46	0.07^
Composite z-scores					
NMCs	0.01 ± 0.61	−0.23 ± 0.7	0.26 ± 0.63	8.22	0.006^
EMCs	−0.04 ± 0.73	−0.41 ± 0.68	0.46 ± 0.74	23.85	<0.0001^
Amyloid imaging					
FBB SUVR	1.34 ± 0.25	1.55 ± 0.14	1.11 ± 0.07	251.47	<0.0001^

Data are presented as mean ± SD; * Determined by Chi-square test; ° Determined by Student’s t test; ^ Determined by One-Way anova. Neuropsychological scores are based on age- and education-adjusted norms obtained from a prior validation study

Aß+= MCI subjects with Aß burden below the threshold of SUVR>1.3; Aß−= MCI subjects with Aß burden below the threshold of SUVR<=1.3; CDR = Clinical Dementia Rating; EMCs = Episodic Memory composite score; NMCs = Non-memory cognition composite score. FBB = [^18^F]Florbetaben. SUVr = Standardized uptake value ratio

Thirty-four out of 63 (54%) aMCI subjects were classified as Aß+, whereas 29 of 63 (46%) were identified as Aß-. The difference between SUVr means of two amyloid clusters, reported in Table 1, was significant (mean ± sd, 1.55 ± 0.14 vs 1.11 ± 0.07; F = 251.5; *P* < 0.0001).

**Fig. 1:**
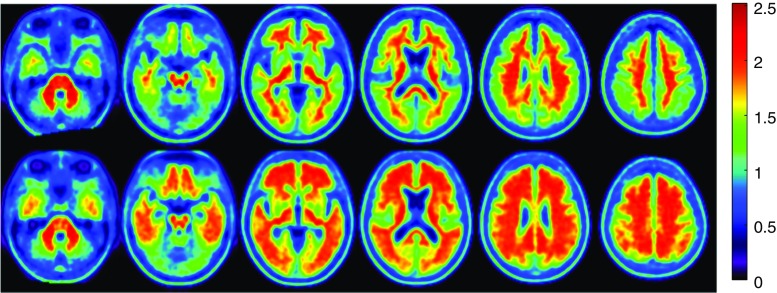
Axial view of [18F]Florbetaben PET amyloid load in mild cognitive impairment. Average axial slices of mild cognitive impairment subjects with low (Aß-; top panel) and high amyloid load (Aß+; bottom panel). Subjects were classified using k-means clustering. Signal intensity is significantly lower in the grey matter regions of the top images compared to those of the bottom images (*P* < 0.0001)

The mean age of Aß + patients was significantly higher compared to Aß- patients (*P* = 0.01); however, no significant correlation was found between amyloid deposition and patient age. No significant differences were found among Aß- and Aß + groups for gender and education (Table 1). Figure 1 shows the representative image of Aß + and Aß- groups calculated by averaging all PET images obtained from subjects above and below the threshold of SUVr = 1.30, respectively.

As reported in Table 1 MMSE and CDR scores did not differ significantly between Aß + and Aß- clusters. By contrast, the results of all other tests worsened in Aß + patients. However, the difference between Aß + and Aß- groups reached the levels of statistical significance only for RFCTc, RCFTd, PR, CVF and DS (Table 1).

Aß + subjects had lower EMCs and NMCs values than Aß- ones, with a greater decrease in EMCs than NMCs scores (Table 1). Differences between mean EMCs and NMCs score in Aß + and Aß- subjects were assessed by one-way ANOVA. Analysis of results showed a significant difference of EMCs (−0.41 ± 0.68 vs. 0.46 ± 0.74, *P* < 0.0001) and NMCs (−0.23 ± 0.72 vs. 0.26 ± 0.63, *P* = 0.006; mean ± sd, *P*) scores between groups of Aß + and Aß- individuals.

Among 35 subjects with episodic memory impairment 28 (80%) had positive amyloid scan and seven (20%) were classified as amyloid negative while in EMCs- group 6 (21%) were Aß + and 22 (79%) were Aß- (OR = 14.67).

Aß positivity significantly identified individuals with episodic memory impairment with a SS of 80% and a SP of 79%, as compared to Aß- subjects (χ2 = 21.48; *P* < 0.00001). The PPV was 82% and NPV was 76% (Table 2).

**Table 2:** Sensitivity and specificity of amyloid accumulation on cognitive decline

Cognitive status	Aß+	Aß−	Chi square	*P*	Sensitivity (95%CI)	Specificity (95%CI)	OR (95%CI)	PPV (95%CI)	NPV (95%CI)
EMCs+	28 (80%)	7 (20%)							
EMCs-	6 (21%)	22 (79%)	21.48	<0.00001	80% (67–93)	79% (63–94)	14.67 (4.3–49.9)	82% (70–95)	76% (60–91)
NMCs+	23 (72%)	9 (28%)							
NMCs-	11 (35%)	20 (65%)	8.39	0.0038	72% (56–87)	65% (48–81)	4.65 (1.6–13.5)	68% (52–83)	69% (52–86)
aMCI+	18 (72%)	7 (28%)							
aMCI-	16 (42%)	22 (58%)	5.42	0.0199	72% (54–90)	58% (42–74)	3.54 (1.2–10.5)	53% (36–70)	76% (60–91)

Aß+= MCI subjects with Aß burden below the threshold of SUVR>1.3; Aß−= MCI subjects with Aß burden below the threshold of SUVR<=1.3; EMCs+ = MCI subjects with episodic memory composite score < = 0.125; EMCs- = MCI subjects with episodic memory composite score > 0.125; NMCs+ = MCI subjects with Non-memory composite score < = 0.06; NMCs- = MCI subjects with Non- memory composite score > 0.06; aMCI+ = MCI subjects with MMSE score < = 24; aMCI- = MCI subjects with MMSE score > 24; CI = confidence interval; OR = Odd ratio; PPV = positive predictive value; NPV = negative predictive value;

Of 32 with non-memory cognition impairment 23 (72%) were Aß + and 9 (28%) were Aß-, whereas in NMCs- group 11 (35%) were Aß + and 20 (65%) were Aß- (OR = 4.65). PET amyloid positivity was associated to non-memory cognition impairment with a SS of 72% and a SP of 65% (χ2 = 8.39; *P* = 0.0038), with a PPV of 68% and a NPV of 69% (Table 2).

Among 25 with global performance impairment 18 (72%) were Aß + and 7 (28%) were Aß-, whereas in MCI- group 16 (42%) were Aß + and 22 (58%) were Aß- (SS = 72%, SP = 58%, PPV = 53%, NPV = 76%, OR = 3.54; χ2 = 5.42; *P* = 0.019) (Table 2).

**Fig. 2:**
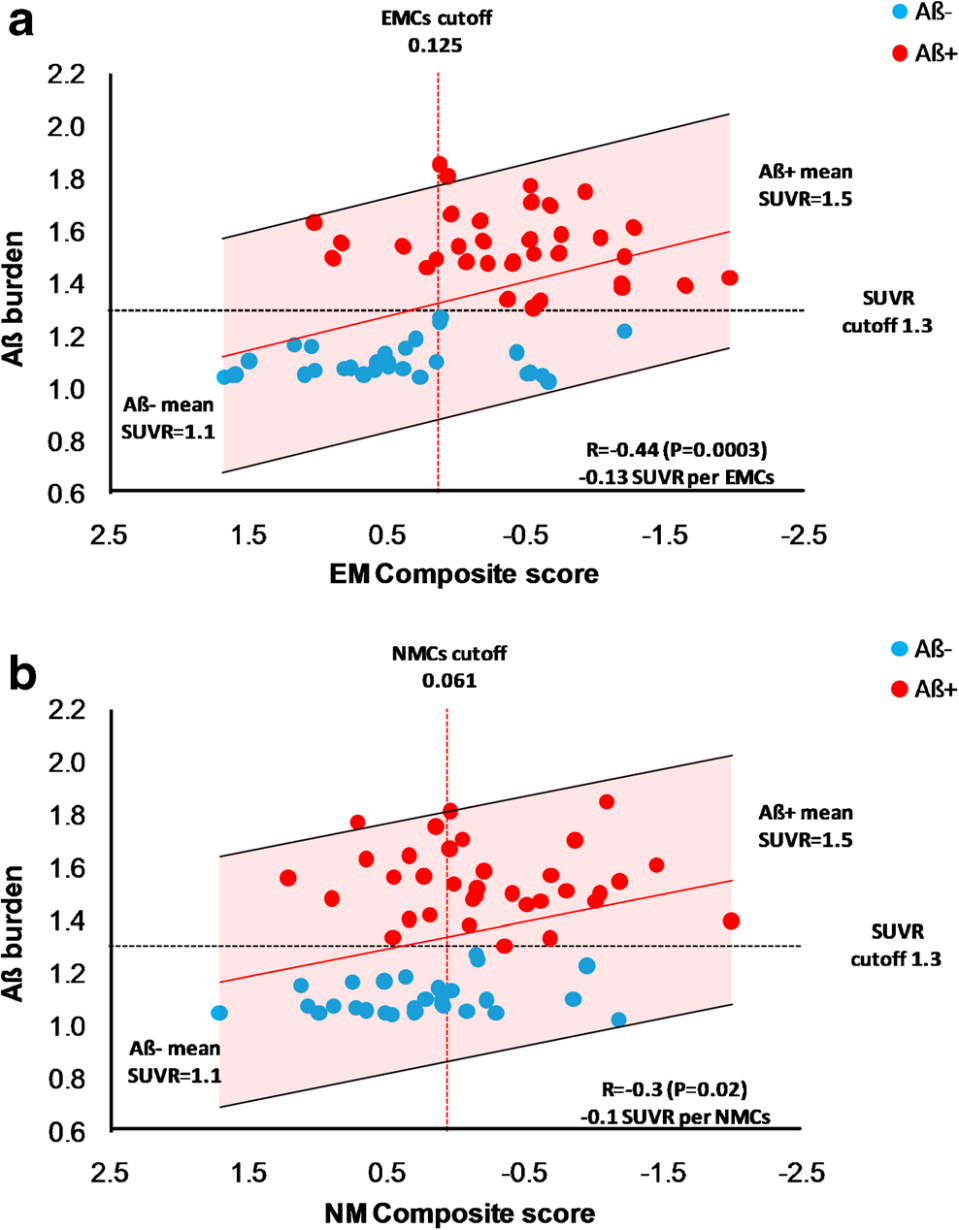
Distribution of individual SUVR values as function of composite score for episodic memory (panel **a**) and non-memory domain (panel **b**). Aβ- and Aβ + MCI subjects are showed as light blue and red circles, respectively. Regression line is showed in red. The black dashed line represents the SUVR cutoff of 1.3 discriminating subjects with high and low tracer retention. The vertical red dashed line represents the 10th percentile of individuals with positive composite score for memory and non-memory cognition tests. Shaded area represents confidence interval of the fit. Aβ = amyloid β. EMCs = episodic memory composite score. NMCs = non-memory composite score. MCI = mild cognitive impairment

The linear regression of individual SUVr values as function of EMCs and NMCs scores is shown in Fig. 2. The analysis confirmed the inverse relationship between Aß burden and both EMCs and NMCs (Fig. 2).

Interestingly, EMCs showed a stronger correlation with Aß burden (*R* = −0.44; *P* = 0.0003) than NMCs (*R* = −0.30; *P* = 0.02) (Fig. 2).

The computed rate of deposition was estimated to be 0.13 SUVr and 0.10 SUVr per Z-transformed ECMs and NMCs unit (Fig. 2).

The significance of the correlation between amyloid retention levels and episodic memory survived also by using in the analysis the tracer retention levels measured in individual brain lobes (Frontal: *R* = −41, *P* = 0.0009, Parietal: *R* = −39, *P* = 0.0015; Temporal: *R* = −45, *P* = 0.0003; Occipital: *R* = −0.48; *P* = 0.0001; Posterior cingulate: *R* = −0.37, *P* = 0.029).

When correlation between amyloid retention in individual brain lobes and non-memory cognition was evaluated, the association showed a statistically significant lower extent than that measured for EMCs (Frontal: *R* = −30, *P* = 0.016; Parietal: *R* = −26, *P* = 0.037; Temporal: *R* = −29, *P* = 0.022; Occipital: *R* = −25, *P* = 0.049; Posterior cingulate: *R* = −0.24, *P* = 0.055).

## Discussion

In recent years several studies have evidenced the important role of PET imaging assessments [11, 30–32] in providing in vivo measurements of brain amyloid deposition levels, confirming significantly higher tracer retention in neocortical areas among MCI subjects who progress to AD compared to subjects who remain stable [7, 33]. Moreover, studies specifically including subjects with aMCI and healthy controls have reported conversion rates from MCI to AD between 59% [33] and 82% among the amyloid positive patients [7].

Further studies have also attempted to define thresholds of amyloid deposition and SURV cut-off values to discriminate cognitively normal subjects, with presumably low amyloid retention levels, from cognitively impaired patients in whom high amyloid retention is expected [9, 31, 34]. So far, however, cut-offs are inconsistent across studies, yielding higher values when studies included healthy controls, cognitive impaired subjects and AD patients (SUVr = 1.6) [24], as compared to studies including MCI subjects alone (SUVr = 1.5) [10, 31].

Hence, our study aimed to identify the beginning signs of amyloid accumulation in subjects belonging to an homogenous clinical entity of aMCI subjects and to evaluate potential association with clinical signs of cognitive decline. By means of [^18^F]Florbetaben PET imaging we were able to discriminate two aMCI subgroups with significantly different Aß retention levels. The elevated levels of FBB uptake were found to be associated to cognitive decline, in particular to a significantly greater decline in episodic memory. Evidence pointed to a threshold of SUVr = 1.30, which was able to divide our cohort into two subpopulations of Aß- (*n* = 29) and Aß + (*n* = 34) with significantly different levels of Aß retention, thus identifying MCI subjects with episodic memory impairment which might progress from MCI to advanced stages of dementia and who most benefit from a closer clinical follow-up or anti-amyloid treatment.

This 1.30 cut-off identified a 54% proportion of Aß + cases which is consistent with the prevalence of AD neuropathology in stable MCI and in those progressing to AD [35, 36] and to the 54% proportion reported by Jansen et al. in MCI subjects with the same average age as that enrolled in this study (75 years) [37]. Likewise, the mean value of amyloid positive subjects in our cohort, which is consistent with baseline value measured in a group of subjects converted from MCI to AD over 3 years [7].

Previous works reported that impairment of episodic memory domain is the most suitable marker for conversion to early AD [24, 38, 39]. As explained by Coulter et al. in a longitudinal study comparing cognitive changes over time in converter and non-converter amnestic MCIs into Alzheimer’s disease, the individuals progressing to AD show abnormal cognition as early as 2 years prior to the diagnosis of dementia, with episodic memory, one of the most affected domains– declining in an almost linear fashion [40].

In our study, the mean cortical florbetaben SUVr was associated with episodic memory decline. Amyloid positivity correctly identified individuals with memory impairment featuring both high sensitivity and specificity (PPV = 82%, NPV = 76%, *P* < 0.00001). Moreover, the amyloid deposition changed linearly with memory decline (episodic memory resulting one of the most affected cognitive domains) with an average increase of 0.13 SUVr per memory composite score. Such observations are in line with previous studies that have reported the linear increase of amyloid deposition in the time interval between the detection of amyloid positivity and the achievement of the average SUVr threshold expected in AD (SUVr>2) [41, 42].

Interestingly, the work of Jack et al. has evidenced a bimodal trend of the deposition curve as function of time. While this was confirmed also in our study, our observations suggest a closer association between increase in amyloid deposition and clinical decline, with the amyloid deposition rate appearing to be a function of episodic memory decline. From a clinical point of view such finding is quite relevant, as it prospects the possibility of (i) reaching an earlier recognition of subjects at risk of progression to AD before neurodegeneration and irreversible related symptoms incur, and (ii) evaluating the efficacy of targeted pharmacological treatments on such patients.

The major limitation of the study lies in the cross-sectional design that does not allow to draw definitive conclusions from this analysis. Our findings should be confirmed by further studies based on longitudinal design.

Another limitation of the present study is that we used CT images for tissue segmentation which is likely to be less accurate than MR-based segmentation. We have chosen this approach on the basis of the recent report on a relatively slight differences between the probability maps of brain tissues obtained from CT and MR images [26]. Comparing 11C-PIB-PET SUV values obtained by correcting partial volume effects with CT and MR-derived probability maps authors did not find significant differences between SUV estimates as shown by the high correlation coefficient reported (R2 = 0.89).

Moreover, if these results were confirmed by other studies, PET based amyloid burden estimates could be assessed also without MR imaging, thus minimizing the patients diagnostic work-up.

In conclusion the present study reports the use of amyloid load as assessed by FBB-PET as a valid approach for objectively dichotomizing MCI individuals in amyloid positive and negative and identifying neuropsychological phenotypes characterized by increased risk of progression to Alzheimer’s disease. Our results have evidenced a significantly greater episodic memory decline in amyloid positive subjects featuring a linear correlation between amyloid load and memory decline in MCI with an average increase of 0.13 SUVr per score of episodic memory decline.
